# Idebenone Decreases Aβ Pathology by Modulating RAGE/Caspase-3 Signaling and the Aβ Degradation Enzyme NEP in a Mouse Model of AD

**DOI:** 10.3390/biology10090938

**Published:** 2021-09-19

**Authors:** Hyun-ju Lee, Ha-Ram Jeong, Jin-Hee Park, Hyang-Sook Hoe

**Affiliations:** 1 Department of Neural Development and Disease, Korea Brain Research Institute (KBRI), 61 Cheomdan-ro, Dong-gu, Daegu 41068, Korea; hjlee@kbri.re.kr (H.-j.L.); hrj21@kbri.re.kr (H.-R.J.); mingmeng1005@kbri.re.kr (J.-H.P.); 2 Department of Brain and Cognitive Sciences, Daegu Gyeongbuk Institute of Science & Technology, Daegu 42988, Korea

**Keywords:** Alzheimer’s disease, idebenone, Aβ, ADAM17, NEP, 5xFAD

## Abstract

**Simple Summary:**

The present study reveals that the FDA-approved drug idebenone has therapeutic effects on the pathology of Alzheimer’s disease (AD) in a mouse model. In particular, idebenone regulates pathological progression associated with Aβ by downregulating the non-amyloidogenic pathway, inhibiting RAGE/caspase-3 signaling, and enhancing Aβ catabolism. In addition, idebenone modulates tauopathy by reducing levels of the tau kinase p-GSK3β, thereby suppressing tau hyperphosphorylation at Thr231. These data suggest that idebenone modulates Aβ and tau pathology in a mouse model of AD.

**Abstract:**

The coenzyme Q10 analogue idebenone is an FDA-approved antioxidant that can cross the blood–brain barrier (BBB). The effects of idebenone on the pathology of Alzheimer’s disease (AD) and the underlying molecular mechanisms have not been comprehensively investigated. Here, we examined the impact of idebenone treatment on AD pathology in 5xFAD mice, a model of AD. Idebenone significantly downregulated Aβ plaque number via multi-directional pathways in this model. Specifically, idebenone reduced the RAGE/caspase-3 signaling pathway and increased levels of the Aβ degradation enzyme NEP and α-secretase ADAM17 in 5xFAD mice. Importantly, idebenone significantly suppressed tau kinase p-GSK3β^Y216^ levels, thereby inhibiting tau hyperphosphorylation at Thr231 and total tau levels in 5xFAD mice. Taken together, the present study indicates that idebenone modulates amyloidopathy and tauopathy in 5xFAD mice, suggesting therapeutic potential for AD.

## 1. Introduction

Alzheimer’s disease (AD) is an irreversible form of dementia characterized by the deposition of amyloid beta (Aβ) through abnormal catabolism of amyloid precursor protein (APP) and the formation of neurofibrillary tangles (NFTs) of hyperphosphorylated tau [1]. APP is predominantly expressed in neuronal synapses and acts as a transmembrane receptor [2]. Nonamyloidogenic sequential proteolytic processing cleaves APP into secreted APPα (sAPPα) via the action of α-secretases (e.g., ADAM10 and ADAM17) and γ-secretase. sAPPα is involved in normal synaptic transmission, dendritic spine formation, synaptic plasticity, and cognitive function [3,4,5]. Genetic modulation of the expression or activity of α-secretases has been shown to alter the levels of cleavage products of APP processing as well as Aβ production. For instance, knockdown of ADAM10 decreases sAPPα levels in mouse primary cortical neuronal cells [6], and in HEK293 APPswe cells, L-isoaspartyl O-methyltransferase knockdown decreases ADAM10/17 expression, thereby enhancing Aβ40 levels [7]. In addition, glutamatergic neuronal activity is suppressed in ADAM17 knockout mice [8]. α-Secretases also cleave thioredoxin-1 (Trx1) to Trx80, which suppresses Aβ aggregation and is reduced in the brains of AD patients [9]. Given the apparent neuroprotective role of α-secretases in AD pathoprogression, upregulating these enzymes is a promising strategy for the prevention and/or treatment of AD [10].

In contrast to the positive effects of α-secretases on AD pathology, sequential cleavage of APP by β-secretase and γ-secretase produces sAPPβ [4], which aggregates into Aβ oligomers that ultimately form the cytotoxic amyloid plaques that are a major hallmark of AD. Receptors for advanced glycation end products (RAGE) accelerate amyloidopathy by transporting Aβ across the blood–brain barrier (BBB) and upregulating β-secretase and γ-secretase activity [11,12]. Therefore, the regulation of Aβ metabolism is also a plausible therapeutic strategy for inhibiting AD pathological progression.

The FDA-approved drug idebenone is a coenzyme Q10 analogue that can cross the BBB [13]. Idebenone has been shown to restore Aβ-induced mitochondrial dysfunction and neural toxicity in primary cortical neurons [14]. In addition, idebenone improves hippocampal-dependent spatial memory function in Aβ-injected rats and mice by promoting Aβ-degrading enzyme levels and antioxidant activity [14,15]. However, whether idebenone affects Aβ plaque deposition and its underlying molecular mechanisms has not been fully investigated in a genetically modified mouse model of AD.

Here, we explored the effect of idebenone on Aβ pathology in 5xFAD mice, which genetically overexpress human APP. We found that idebenone inhibited Aβ plaque accumulation by upregulating the α-secretase ADAM17 and the Aβ degradation enzyme NEP and suppressing RAGE/cleaved caspase-3 signaling in 5xFAD mice. In addition, idebenone modulated tau phosphorylation at Thr231 by inhibiting the tau kinase GSK-3β in 5xFAD mice. Taken together, the present findings indicate that idebenone modulates AD pathology and thus may have a therapeutic effect on neurodegenerative diseases.

## 2. Materials and Methods

### 2.1. 5xFAD Mice

To examine the effects of idebenone on AD pathology (i.e., Aβ, tau), male 5xFAD transgenic mice (MMRRC Stock 34848; B6.Cg-Tg (APPSwFlLon, PSEN1*M146L*L286V)6799Vas/Mmjax; Jackson Laboratory, Bar Harbor, ME, USA) were used as a mouse model of AD. These mice overexpress two transgenes carrying five familial AD mutations in human APP (Swedish mutation K670N, M671L; Florida mutation I716V; London mutation V717I) and human presenilin 1 (PSEN1 M146L, L286V) under transcriptional control by the Thy1 promoter. Genotyping was performed by RT-PCR analysis of tail genomic DNA to detect the human *APP* and *PSEN* genes. For in vivo experiments, idebenone (100 mg/kg, i.p.) or vehicle (5% DMSO + 10% PEG + 20% Tween80) was administered to 3-month-old mice daily for 14 days prior to immunofluorescent staining.

### 2.2. Idebenone Injection

Idebenone was purchased from TCI (Tokyo, Japan, cat number: I0848) and dissolved in 5% DMSO, 10% polyethylene glycol (PEG) and 20% Tween 80 for intraperitoneal (i.p.) administration at 100 mg/kg daily for 14 days.

### 2.3. Immunofluorescent Staining

5xFAD mouse brains were sequentially fixed overnight in 4% paraformaldehyde at 4 °C and for 2 days in 30% sucrose solution at 4 °C. A cryostat (Leica CM1850, Wetzlar, Germany) was used to obtain 35-µm-thick coronal slices, which were then permeabilized in 0.2% Triton X-100 and 10% normal goat serum in PBS for 1 h at room temperature (RT). Next, immunostaining was performed with anti-6E10, anti-RAGE, anti-cleaved caspase-1, anti-NEP, anti-IDE, anti-ADAM17, anti-ADAM10, anti-AT180 (to detect tau phosphorylation at Thr231), anti-Tau-5 (to detect total tau levels), anti-p-GSK3β, anti-p-CDK5, or anti-DYRK1A for 24–48 h at 4 °C, followed by the corresponding secondary antibody at RT. Images were acquired by fluorescence microscopy (DMi8, Leica Microsystems, Wetzlar, Germany) and analyzed by ImageJ (version 1.53a, US National Institutes of Health, Bethesda, MD, USA). Table 1 provides detailed information on the primary and secondary antibodies.

### 2.4. Statistics

GraphPad Prism 7 software (GraphPad Software, San Diego, CA, USA) was used to generate graphs and for statistical analysis. Data are presented as individual data points and the mean ± SEM. Comparisons between two groups were performed using an unpaired two-tailed Student’s *t*-test. Asterisks indicate significance: * *p* < 0.05, ** *p* < 0.01, and *** *p* <  0.001.

## 3. Results

### 3.1. Idebenone Reduces Aβ Plaque Number in 5xFAD Mice

**Figure 1:**
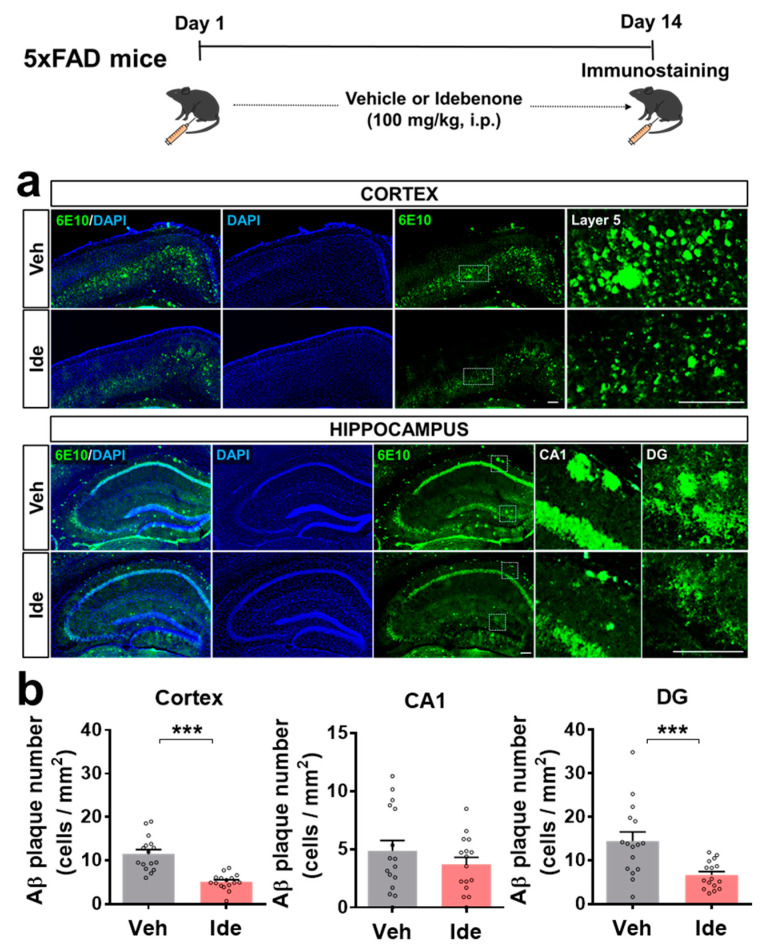
Idebenone significantly decreases Aβ pathology in a mouse model of AD. (**a**) Representative images of cortical and hippocampal 6E10 immunofluorescent staining. Idebenone or vehicle was administered daily to 3-month-old mice for 14 consecutive days, as shown at the top of the figure. Immunostaining of brain sections with anti-6E10 was subsequently performed. (**b**) Quantification of the results (n = 16 brain slices from four mice/group). *** *p* < 0.001 vs. vehicle-treated control. Scale bar = 200 μm.

To examine the effects of idebenone on Aβ pathology, 5xFAD mice were injected with idebenone (100 mg/kg, i.p.) or vehicle daily for 2 weeks, and brain slices were immunostained with anti-6E10. Interestingly, the number of Aβ plaques in the cortex and hippocampal DG region was significantly reduced in idebenone-treated 5xFAD mice (Figure 1a,b).

However, idebenone did not alter Aβ plaque number in the hippocampal CA1 region (Figure 1a,b). These data suggest that idebenone selectively modulates Aβ pathology in regions of the brain in this mouse model of AD.

### 3.2. Idebenone Decreases RAGE and Caspase-3 Levels in 5xFAD Mice

**Figure 2:**
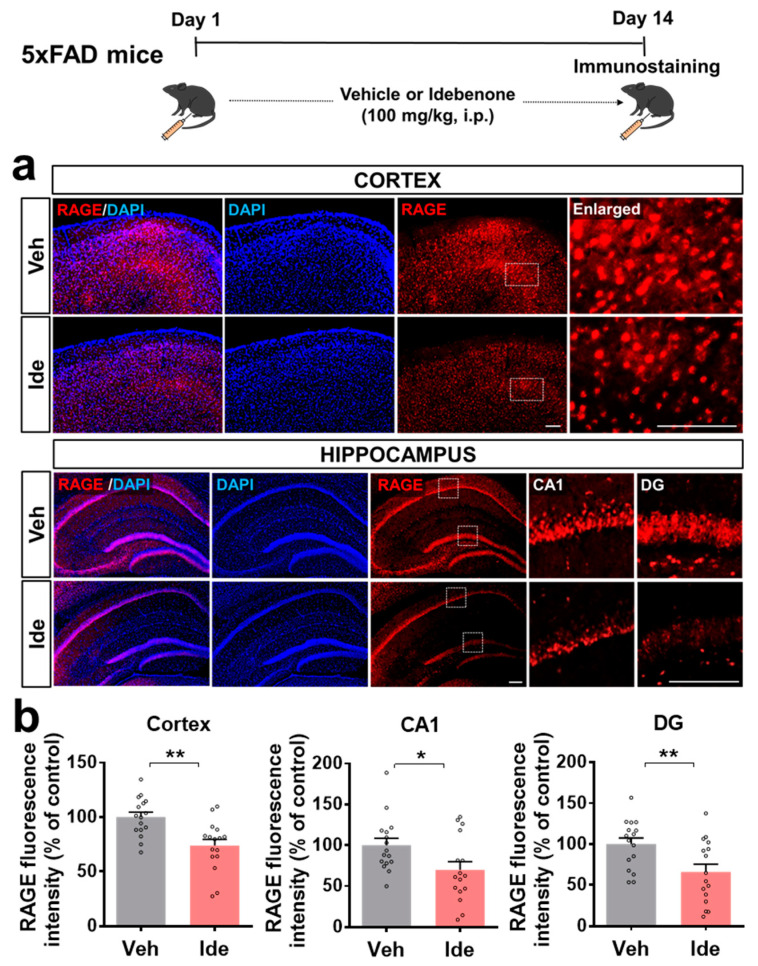
Idebenone significantly reduces RAGE expression in 5xFAD mice. (**a**) Representative images of cortical and hippocampal RAGE immunofluorescent staining. Idebenone or vehicle was administered daily to 3-month-old mice for 14 consecutive days as shown at the top of the figure. Immunostaining of brain sections with anti-RAGE was then performed. (**b**) Quantification of the results (n = 16 brain slices from four mice/group). * *p* < 0.05 and ** *p* < 0.01 vs. vehicle-treated control. Scale bar = 100 μm for cortex and 200 μm for hippocampus.

We next investigated the molecular mechanisms underlying the effects of idebenone on Aβ plaque number. In vivo experiments were performed according to the paradigm in Figure 1, and immunofluorescent staining was performed with an antibody against RAGE, which is known to regulate Aβ metabolism. We observed that idebenone treatment of 5xFAD mice significantly downregulated cortical and hippocampal RAGE levels (Figure 2a,b).

**Figure 3:**
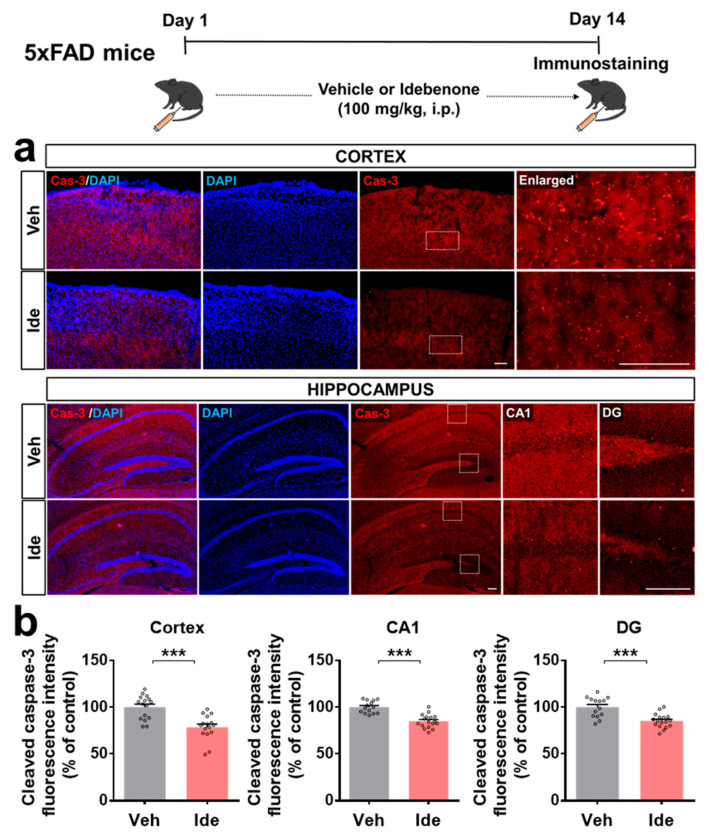
Idebenone significantly suppresses cleaved caspase-3 levels in a mouse model of AD. (**a**) Representative images of cleaved caspase-3 immunofluorescent staining in the cortex and hippocampus. Idebenone or vehicle was administered daily to 3-month-old mice for 14 consecutive days as shown at the top of the figure. Immunostaining of brain sections with anti-caspase-3 was then performed. (**b**) Quantification of the results (n = 14–17 brain slices from 4 mice/group). *** *p* < 0.001 vs. vehicle-treated control. Scale bar = 100 μm for cortex and 200 μm for hippocampus.

We then investigated whether idebenone alters the expression of the downstream signaling molecule caspase-3 in 5xFAD mice. Importantly, idebenone significantly suppressed Aβ-mediated caspase-3 levels (Figure 3a,b). These data indicate that idebenone reduces Aβ plaque number by modulating RAGE/caspase-3 signaling in this model of AD.

### 3.3. Idebenone Upregulates the Expression of the Aβ Degradation Enzyme NEP in 5xFAD Mice

**Figure 4:**
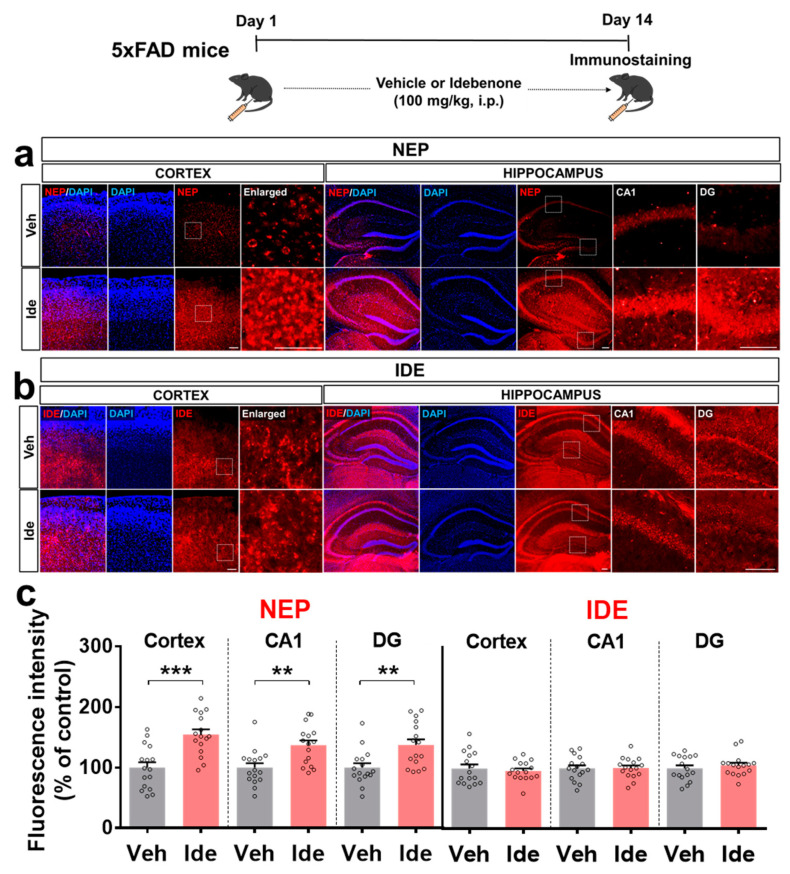
Idebenone significantly upregulates NEP levels in a mouse model of AD. (**a,b**) Representative images of cortical and hippocampal NEP and IDE immunofluorescent staining. Idebenone or vehicle was administered daily to 3-month-old mice for 14 consecutive days, as shown at the top of the figure. Immunostaining of brain sections was then performed with anti-NEP and anti-IDE. (**c**) Quantification of the results (n = 16 brain slices from four mice/group). ** *p* < 0.01 and *** *p* < 0.001 vs. vehicle-treated control. Scale bar = 100 μm for cortex and 200 μm for hippocampus.

To test whether idebenone alters Aβ pathology by impacting Aβ degradation enzymes, immunofluorescent staining of brain slices from 5xFAD mice was performed with antibodies against the Aβ degradation enzymes, neprilysin (NEP), and the insulin-degrading enzyme (IDE). Idebenone treatment significantly increased cortical and hippocampal NEP levels (Figure 4a,c) but had no significant effect on IDE levels (Figure 4b,c). These data suggest that idebenone selectively regulates Aβ degradation enzymes to alter Aβ pathology in 5xFAD mice.

### 3.4. Idebenone Upregulates the Expression of the α-Secretase ADAM17 in 5xFAD Mice

**Figure 5:**
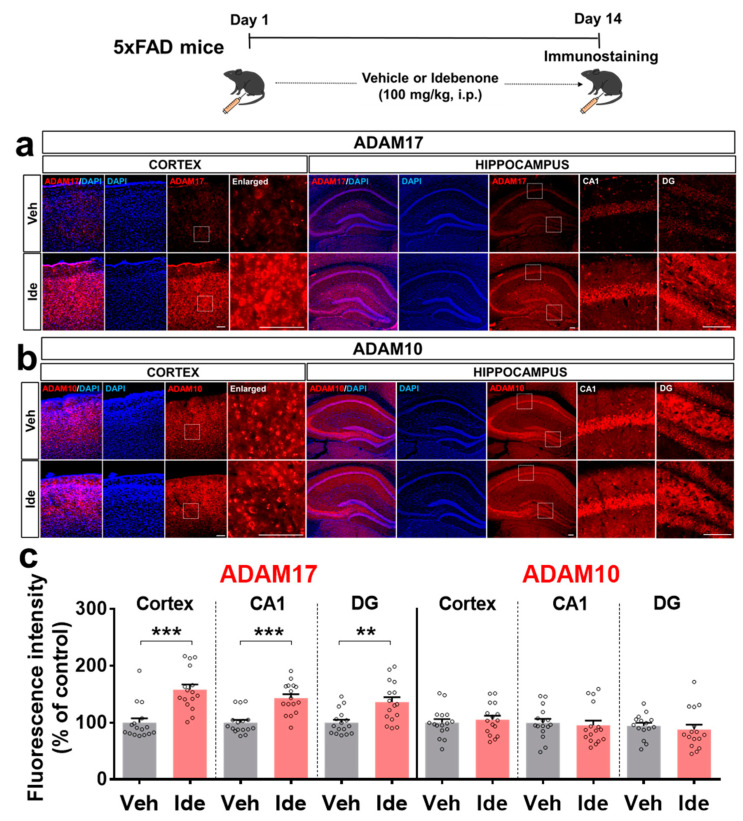
Idebenone significantly increases ADAM17 levels in a mouse model of AD. (**a,b**) Representative images of cortical and hippocampal ADAM17 and ADAM10 immunofluorescent staining. Idebenone or vehicle was administered daily to 3-month-old mice for 14 consecutive days as shown at the top of the figure. Immunostaining of brain sections was then performed with anti-ADAM17 and anti-ADAM10. (**c**) Quantification of the results (n = 15–16 brain slices from 4 mice/group). ** *p* < 0.01 and *** *p* < 0.001 vs. vehicle-treated control. Scale bar = 100 μm for cortex and 200 μm for hippocampus.

To further determine how idebenone regulates Aβ pathology *in this model of AD*, we investigated the effects of idebenone on α-secretase expression. For these experiments, 5xFAD mice were injected *daily* with vehicle or idebenone (100 mg/kg, i.p.) for 2 weeks, and immunostaining was conducted with anti-ADAM10 and anti-ADAM17. Interestingly, idebenone significantly enhanced the expression of ADAM17 but not ADAM10 (Figure 5a–c), suggesting that idebenone differentially regulates *α*-secretase enzyme expression to alter Aβ pathology.

### 3.5. Idebenone Decreases Tau Phosphorylation at Thr231 (AT180) and p-GSK3β Levels in 5xFAD Mice

Given the effects of idebenone on Aβ pathology via the alteration of RAGE/caspase-3 signaling and the Aβ degradation enzyme NEP in 5xFAD mice, we examined whether idebenone modulates tau pathology. To test this, mice were injected with vehicle or idebenone (100 mg/kg, i.p.) daily for 2 weeks, and immunostaining was conducted with anti-AT180 and anti-tau-5. Idebenone treatment significantly downregulated AT180 immunoreactivity in the hippocampal CA1 region in 5xFAD mice, whereas no changes were observed in the cortex and hippocampal DG region (Supplementary Figure S1a,d). Surprisingly, we also found that idebenone treatment significantly reduced total tau levels in the hippocampus, but not the cortex in 5xFAD mice (Supplementary Figure S1b,d).

We then examined the effects of idebenone on tau kinases in 5xFAD mice. Idebenone significantly reduced hippocampal but not cortical levels of tau kinase p-GSK3β^Y216^ (Supplementary Figure S1c,d). In addition, idebenone did not alter levels of the tau kinases p-CDK5 and DYRK1A in the brain (Supplementary Figures S2 and S3). These data indicate that idebenone differentially modulates tau kinases to alter tau pathology.

## 4. Discussion

Cytotoxic Aβ plaque deposition contributes to the pathological progression of AD [16,17]. Here, we revealed that idebenone administration significantly ameliorates Aβ plaque accumulation in 5xFAD mice by enhancing levels of the α-secretase ADAM17 and Aβ degradation enzyme NEP, and inhibiting the amyloidogenic RAGE/cleaved caspase-3 signaling pathway. We further found that idebenone downregulates tau phosphorylation at Thr231 by suppressing tau kinase GSK-3β in 5xFAD mice. These findings support the plausibility of therapeutic effects of idebenone on neurodegenerative diseases, including AD.

AD patients exhibit increased hippocampal neuronal RAGE intensity and protein levels compared with elderly controls [18]. In addition, 3xTg mice, a transgenic mouse model of AD, show increased neuronal, microglial, and astrocytic RAGE expressions in the brain compared with WT mice [19]. In Tg-mAPP mice, intracellular Aβ and RAGE are co-localized in hippocampal pyramidal neurons, and RAGE accelerates Aβ-induced cytotoxicity via translocation of extracellular Aβ to the intraneuronal space [20]. The administration of Aβ_1-40_ peptide in rats exaggerates neuronal dysfunction by upregulating the RAGE-P38-caspase-3 pathway [21]. Idebenone is an analogue of coenzyme Q10. Coenzyme Q10 reduces RAGE levels in the joint tissue of rats with osteoarthritis [22], and several studies have found that idebenone inhibits Aβ plaque accumulation in Aβ peptide-infused rats and mice [15,23]. In high-fat-diet-treated human endothelial cells, idebenone pretreatment decreases caspase-3 expression [24]. The present study is the first to examine the effects of idebenone on Aβ plaque number and RAGE/caspase-3 levels in 5xFAD mice. In combination with the literature, our findings suggest that idebenone significantly downregulates Aβ plaque number by modulating RAGE/caspase-3 signaling (Figure 1, Figure 2 and Figure 3). Interestingly, several recent studies have demonstrated that RAGE is closely related to modulation of mitochondrial function. For example, increased interaction of RAGE with AGE results in excessive release of superoxide at mitochondrial complex I in both diabetic rats and rat primary mesangial cells overexpressing RAGE under hyperglycemia [25]. In murine osteoblastic cells, the RAGE-specific inhibitor FPS-ZM1 ameliorates AGE-mediated apoptosis by suppressing mitochondrial ROS production and increasing mitochondrial length and density [26]. Furthermore, Aβ peptide-mediated mitochondrial dysfunction was suppressed in the cortical neurons from RAGE knockout mice [20]. Interestingly, the treatment of synthetic RAGE fragment diminished activity of cortical and hippocampal mitochondrial respiratory enzymes in a sporadic AD mice model [27]. Taken together, these observations raise the possibility that idebenone inhibits the Aβ-RAGE interaction, thereby facilitating mitochondrial function and, in turn, protecting neurons from Aβ-mediated apoptosis.

Enzymatic Aβ degradation is critical for modulating Aβ plaque accumulation [28], and NEP and IDE are major proteases involved in this pathway. NEP enzymatic activity and immunohistochemical intensity in the cortex are significantly lower in AD patients compared with non-AD controls [29]. Furthermore, genetic ablation of NEP upregulates the accumulation of Aβ, reduces spatial/recognition memory, and impairs hippocampal synaptic plasticity in mice [30,31]. IDE-deficient mice also exhibit increases in Aβ deposition and APP intracellular domain levels in the brain [32]. Here, we found that idebenone treatment increased NEP expression in 5xFAD mice, whereas IDE expression was not altered (Figure 4). There are several potential mechanisms by which idebenone may differentially regulate the expression of NEP and IDE in 5xFAD mice. First, NEP may have a greater impact on Aβ degradation than IDE. Although both NEP and IDE are involved in the process of Aβ catabolism, a few studies have indicated that NEP inhibits Aβ deposition more effectively than IDE. For example, nep mRNA levels, NEP protein levels and NEP activity in the frontal cortex are significantly reduced in AD patients, whereas ide mRNA levels are elevated, and IDE protein levels and activity are unchanged [33]. Another study reported that the overexpression of the nep gene reduced Aβ plaque load in APP/PS1 mice, whereas overexpression of the ide gene had no effect [34]. These observations suggest that idebenone modulates NEP expression more sensitively than IDE levels to reduce Aβ accumulation in 5xFAD mice. Second, IDE expression and activity are differentially regulated by drugs [28,35]. Therefore, future studies will explore whether idebenone modulates IDE activity without altering IDE expression in 5xFAD mice. Lastly, two weeks of idebenone treatment might not be sufficient to regulate IDE expression in 5xFAD mice, and thus future work will investigate whether longer idebenone treatment durations affect IDE expression or activity in 5xFAD mice.

Nonamyloidogenic APP processing is also decisive for reducing Aβ plaque load. ADAM17 and ADAM10 are the most critical α-secretases in the anti-amyloidogenic Aβ catabolism pathway [36]. A single rare nonsynonymous variant of ADAM17 results in the loss of function of ADAM17 followed by increased app gene expression in the human brain [37]. Compared with the controls, AD patients have significantly lower cerebrospinal fluid (CSF) ADAM10 levels [38]. We observed that idebenone treatment remarkably enhanced ADAM17 expression in the brain in 5xFAD mice, whereas ADAM10 levels were not altered (Figure 5). N-terminomics approaches have revealed that ADAM17 and ADAM10 have distinct APP cleavage sites [39]. ADAM10 has higher specificity for cleavage at Y303 in the conserved region of the central APP domain, whereas the ADAM10/17 complex has greater specificity for L127 in the N-terminal growth factor-like domain (E2) and V244, I591, and V594 in the E2 domain of APP [39]. Furthermore, ADAM17 exhibits selective shedding activity toward sAPP696, whereas ADAM10 primarily exhibits shedding activity for sAPP751 [39]. Taken together, these observations suggest that idebenone promotes ADAM17-dependent nonamyloidogenic APP cleavage to suppress Aβ plaque deposition.

The aggregation of hyperphosphorylated tau exaggerates AD pathological progression [40,41]. Interestingly, a recent study demonstrated that 10 μM idebenone inhibits tau aggregation in SH-SY5Y cells [40]. However, the influence of idebenone on tauopathy in vivo has not been fully characterized. Our study is the first to show that idebenone can significantly reduce tau phosphorylation at threonine residue 231 (AT180) by inhibiting tau kinase GSK-3β (Y216) (Supplementary Figure S1). Moreover, we observed that hippocampal, but not cortical, tau levels were significantly downregulated in idebenone-treated 5xFAD mice (Supplementary Figure S1). However, levels of the tau kinases DYRK1A and p-CDK5 were not altered in idebenone-treated 5xFAD mice (Supplementary Figures S2 and S3). It is possible that idebenone affects other tau phosphorylation sites to alter tau pathology in 5xFAD mice. Future work will investigate whether idebenone regulates tau phosphorylation at serine/threonine/tyrosine residues located in SP/TP motifs or KXGS motifs in 5xFAD and Tau Tg PS19 mice.

Since memory impairment is a primary symptom of AD development [42], behavioral assessments would be useful for further evaluating the efficacy of idebenone in ameliorating AD pathology. This limitation of the present study will be addressed by assessing the effects of idebenone on cognitive function in mouse models of AD in a future study. In addition, the current study revealed that idebenone effectively regulates Aβ pathophysiology in the early phase of AD represented by 3-month-old 5xFAD mice. Further investigations in aged AD mouse models (e.g., 6-month-old and 12-month-old 5xFAD mice) will be necessary to assess whether idebenone modulates AD pathology, including Aβ accumulation/degradation and memory dysfunction, in moderate and severe stages of AD.

In summary, this study is the first to reveal the effects of idebenone in 5xFAD mice, which genetically overexpress human APP. Idebenone downregulated Aβ plaque deposition by enhancing the expression of Aβ degradation enzymes, increasing non-amyloidogenic APP processing, and suppressing pro-amyloidogenic RAGE/caspase-3 signaling. In addition, idebenone inhibited tau hyperphosphorylation at Thr231 by suppressing GSK-3β^Y216^ expression, suggesting the potential therapeutic utility of idebenone for AD.

## 5. Conclusions

**Figure 6:**
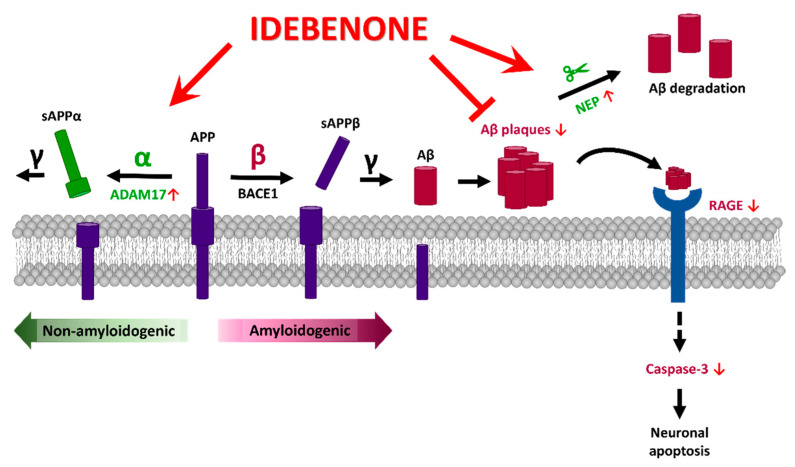
Graphical illustration of the effects of idebenone on AD pathology in the brain in 3-month-old 5xFAD mice. Idebenone regulates Aβ pathogenesis in this early-phase AD mouse model by promoting the expression of the non-amyloidogenic α-secretase ADAM17 and the Aβ degradation enzyme NEP and suppressing the apoptotic RAGE-caspase 3 signaling pathway. These effects support a potential neuroprotective impact of idebenone on Aβ plaque-mediated neuronal apoptosis.

In the present study, we investigated the effects of the FDA-approved antioxidant idebenone on AD pathology in a transgenic mouse model of AD. We found that idebenone inhibited Aβ plaque deposition by suppressing the amyloidogenic RAGE/capase-3 axis and upregulating levels of the Aβ degradation enzyme NEP and α-secretase ADAM17. Moreover, idebenone decreased tau phosphorylation at Thr231 (AT180) and total tau levels by downregulating tau kinase p-GSK3β^Y216^ expression in 5xFAD mice. Collectively, our data demonstrate that idebenone modulates Aβ and tau pathology in a mouse model of AD (Figure 6), suggesting that idebenone may be useful for treating neurodegenerative diseases.

## Figures and Tables

**Table 1:** Primary antibodies.

Immunogen	Host Species	Dilution	Manufacturer	Catalog No.
6E10	Mouse	1:500	BioLegend	803002
RAGE	Rabbit	1:200	Abcam	AB3611
Caspase-3	Rabbit	1:100	Cell Signaling	9664
NEP	Rabbit	1:200	Millipore	AB5458
IDE	Rabbit	1:200	Abcam	AB32216
ADAM17	Rabbit	1:100	Abcam	AB2051
ADAM10	Rabbit	1:100	Abcam	AB1997
AT180	Mouse	1:100	Invitrogen	MN1040
Tau5	Mouse	1:100	Invitrogen	AHB0042
p-GSK-3β^Y216^	Rabbit	1:200	Abcam	AB75745
p-CDK-5	Rabbit	1:200	LSBio	LS-C354604
DYRK1A	Rabbit	1:200	Abcam	AB69811

## Data Availability

The data presented in this study are available in the article and the Supplementary Material.

## Supplementary Materials

The following are available online at https://www.mdpi.com/article/10.3390/biology10090938/s1: Figure S1: Idebenone reduces tau phosphorylation at Thr231 (AT180) and tau kinase p-GSK3βY216 levels in a mouse model of AD. Figure S2: Idebenone does not alter tau kinase p-CDK5 levels in a mouse model of AD. Figure S3: Idebenone does not affect tau kinase DYRK1A levels in in a mouse model of AD.
