# Supplementary material for: Idebenone Decreases Aβ Pathology by Modulating RAGE/Caspase-3 Signaling and the Aβ Degradation Enzyme NEP in a Mouse Model of AD
Source: Biology (Basel). 2021 Sep 19;10(9):938. doi: 10.3390/biology10090938 (PMC8471964; doi:10.3390/biology10090938)
Supplement: Supplementary file 1 [file biology-10-00938-s001.zip › biology-1376783-supplementary.pdf]

biology

Supplementary

Idebenone Decreases Aβ Pathology by Modulating RAGE/Caspase-3 Signaling and the Aβ Degradation Enzyme NEP in a Mouse Model of AD

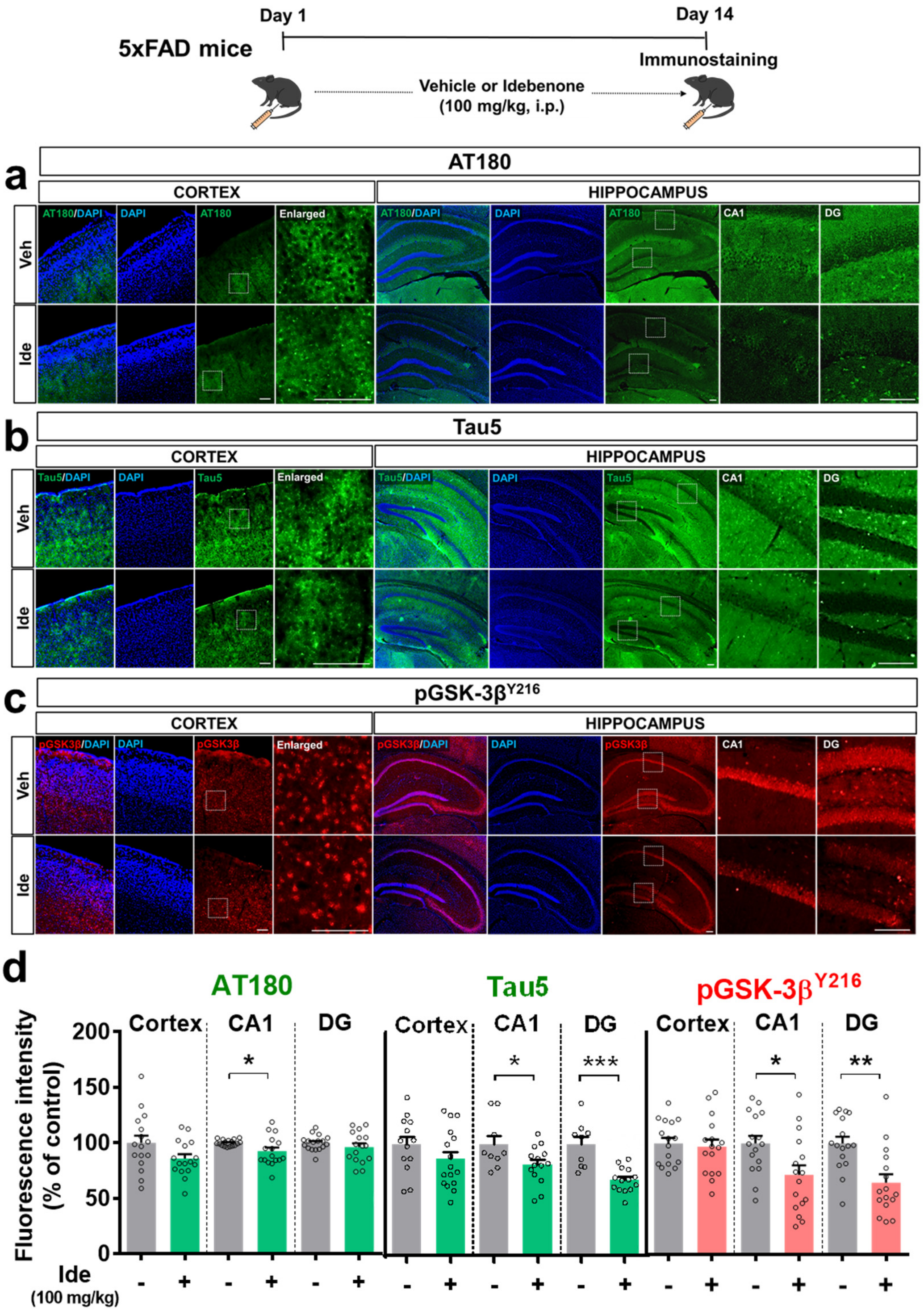

Supplementary Figure S1. Idebenone reduces tau phosphorylation at Thr231 (AT180) and tau kinase p-GSK3β^{Y216} levels in a mouse model of AD. (a, b, c) Representative images of cortical and hippocampal AT180, Tau-5, and p-GSK3β^{Y216} immunofluorescence staining. Idebenone or vehicle was administered daily to 3-month-old 5xFAD mice for 14 consecutive days as shown at the top of the figure. Immunostaining of brain sections with anti-AT180, anti-Tau-5, and anti-p-GSK3β were then performed. (d) Quantification of data from a-c (n = 16–17 brain slices from 4 mice/group for a, c; n = 9–16 brain slices from 3-4 mice/group for b). *p < 0.05, **p < 0.01 and ***p < 0.001 vs. vehicle-treated control. Scale bar = 100 μm for cortex and 200 μm for hippocampus.

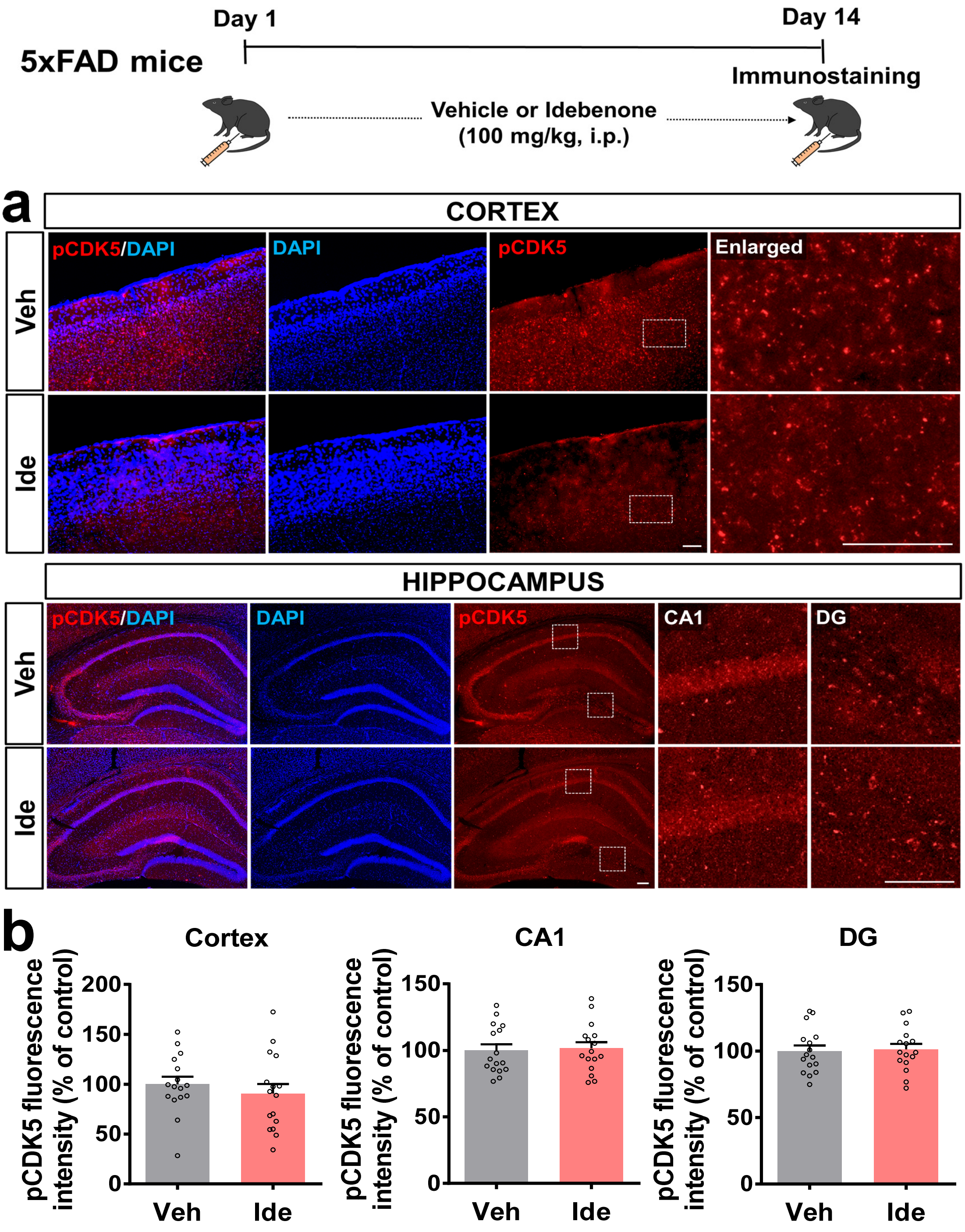

Supplementary Figure S2. Idebenone does not alter tau kinase p-CDK5 levels in a mouse model of AD. (**a**) (**a**) Representative images of cortical and hippocampal p-CDK5 immunofluorescent staining. Idebenone or

vehicle was administered daily to 3-month-old 5xFAD mice for 14 consecutive days as shown at the top of the figure. Immunostaining of brain sections with anti-p-CDK5 was then performed. (**b**) Quantification of data from **a** (n = 16 brain slices from 4 mice/group). Scale bar = 100 μm for cortex and 200 μm for hippocampus.

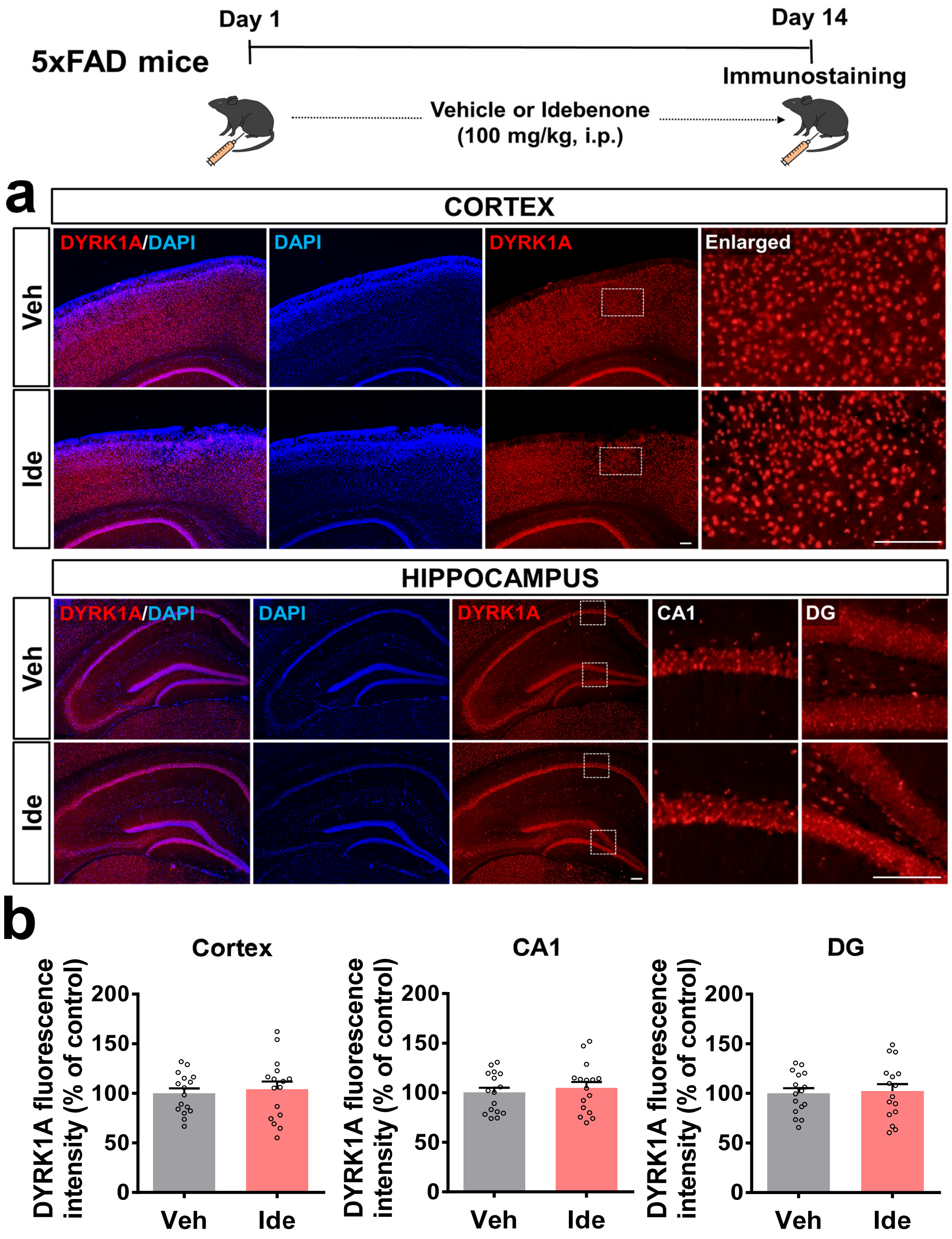

Supplementary Figure S3. Idebenone does not affect tau kinase DYRK1A levels in a mouse model of AD. (**a**) Representative images of cortical and hippocampal DYRK1A immunofluorescent staining. Idebenone or vehicle was administered daily to 3-month-old 5xFAD mice for 14 consecutive days as shown at the top of the figure. Immunostaining of brain sections with anti-DYRK1A was then performed. (**b**) Quantification of data from **a** (n = 16 brain slices from 4 mice/group). Scale bar = 100 μm for cortex and 200 μm for hippocampus.
